# Syndrome of Inappropriate Secretion of Antidiuretic Hormone Cholestasis and Pericardial Effusion Due to Brucellosis Infection: A Case Report

**DOI:** 10.1155/2010/850402

**Published:** 2010-08-09

**Authors:** Ahmet Cumhur Dülger, Özgür Kemik, Aziz Sümer, Hüseyin Akdeniz, Mehmet Emin Küçükoğlu, Esra Turan Canbaz, Veyis Itik, Enver Aytemiz

**Affiliations:** ^1^Department of Gastroenterology, Yüzüncü Yil University School of Medicine, 65200 Van, Turkey; ^2^Department of General Surgery, Yüzüncü Yil University School of Medicine, 65200 Van, Turkey; ^3^Department of Radiology, Yüzüncü Yil University School of Medicine, 65200 Van, Turkey

## Abstract

Syndrome of inappropriate secretion of antidiuretic hormone (SIADH) is an extremely rare complication of infectious diseases. A rare case of brucellosis complicated by syndrome of inappropriate secretion of antidiuretic hormone (SIADH) cholestasis and pericardial involvement is reported. A 27-year-old woman was admitted for fever, abdominal pain, and scleral icterus. Her medical history revealed no recent use of diuretic agents. In addition to cholestasis and elevated liver enzymes, euvolemic hyponatremia, hypouricemia, low plasma osmolality, and high urinary osmolality were also detected. Surrenal and thyroid tests were also within normal range. Echocardiography revealed minimal pericardial effusion with normal cardiac functions. The final diagnosis was SIADH due to Brucellosis. Hyponatremia, cholestasis, and pericardial disease were resolved with effective antibrucellar treatment with streptomycine and doxycycline. After completing treatment of brucellosis, there was not any more evidence of cholestasis and pericardial fluid.

## 1. Introduction

The syndrome of inappropriate secretion of antidiuretic hormone (SIADH) is characterised by euvolemic hyponatremia, low plasma osmolality, high urinary osmolality, elevated urinary natriuresis, hypouricemia, and lack of evidence of other hyponatremic diseases. This syndrome occurs in response to continued antidiuretic hormone (ADH) release in spite of low serum osmolality. Central nervous system disorders, pulmonary tuberculosis, endocrine diseases, paraneoplastic syndromes, and various drugs may cause SIADH [1–3].

Human Brucellosis is caused by Brucella abortus, melitensis, or suis and characterised by recurrent fever, splenomegaly, and lymphadenopathy. This disease is acquired by direct contact with infected animals and by consuming noncooked milk or unpasteurized cheese. Brucellosis can also lead to cholestatic hepatitis and cardiac or pericardiac involvement [4].

Brucellosis is also an endemic and zoonotic disease for Mediterranean basin and Turkey [5, 6].

In this paper, we describe a female patient with fever, cholestasis, pericardial effusion, and SIADH that was caused by brucellosis infection and resolved with antibrucellar therapy.

## 2. Case

A 27-year-old woman was referred to the emergency department with complaints of icterus, nocturnal fever, arthralgia, and abdominal pain. The patient had been well until two weeks earlier, when symptoms developed. She was a farmer in a village and owned a lot of sheep. There were no allergies and no history of recent use of drugs but she was consuming a large amount of nonpasteurized traditional cheese.

On physical examination, the patient was nonedematous. Her temperature was 38.5°C, with a blood pressure of 70/45 mm Hg, a pulse rate of 70 beats per minute, and respirations of 18 per minute. The oxygen saturation was 96 percent while she was breathing room air.

The liver edge was palpable 5 cm below the costovertebral margin. Her right upper quadrant of abdomen was diffusely tender to palpation. Additionally, there was marginal splenomegaly. The heart sounds and the results of the remainder of the examination were normal.

Laboratory studies performed at admission revealed a WBC count of 5400 cells/mm³, a hemoglobin level of 10.6 g/dL, a platelet count of 216.000 platelets/mm³, an aspartate aminotransferase level of 224 U/L, an alanine aminostransferase of 159 U/L, an alkaline phosphatase level of 2340 U/L, a gamma glutamyl transferase level of 597 U/L, a direct bilirubin level of 3.1 mg/dL, an albumin level of 3.3 g/dL, a globulin level of 4.3 g/dL and a uric acid level of 1.4 g/dL. Serum pituitary, thyroid, and adrenal function tests were in normal ranges.

Her other laboratory parameters included erythrocyte sedimentation rate 47 mm/hr, C-Reactive Protein level 26 U/L, serum sodium level 124 mmol/L, potassium 4.4 mmol/L, chloride 95 mmol/L, and plasma osmolality 262 mmol/kg/H2O. Urine biochemistry showed osmolality 274 mmol/kg/H2O and urinary sodium 64 mmol/L. Serologic tests for Epstein-Barr virus, cytomegalovirus, and hepatitis viruses were negative. Electrocardiographic examination showed minimal ST-T elevations.

**Figure 1:**
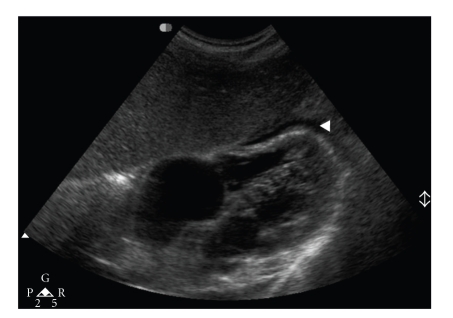
Ultrasonography of abdomen showed a minimal pericardial effusion.

**Figure 2:**
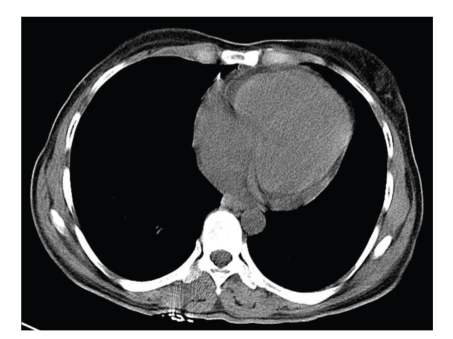
Computed Tomography scan (CT scan) of abdomen showed a minimal pericardial effusion.

Ultrasonography and Computed Tomography scan (CT scan) of the abdomen showed a small pericardial effusion with mild splenomegaly and hepatomegaly (Figures 1 and 2). However, there was no evidence of biliary dilatation or cholecystitis. CT scan of thorax and brain revealed no abnormality. Echocardiography detected mild pericardial effusion without cardiac dysfunction.

At the next day, serum agglutination test for Brucella was positive and its titer was 1/1240. The diagnosis of SIADH in association with cholestatic hepatitis and pericardial effusion due to Brucellosis was made. After intramuscular streptomycin (1000 mg/day for 20 days) with oral Doxycycline (100 mg twice daily for 6 weeks), the patient's symptoms, cholestasis, pericardial effusion, and hyponatremia resolved dramatically.

## 3. Discussion

Here we report a patient who presented with nocturnal fever, leucopenia, cholestasis, and hyponatremia and in whom we made the diagnosis. 

Laboratory testing is always required to establish the diagnosis of SIADH. So, the diagnosis of SIADH in our case was established by (a) euvolemic hyponatremia with concomitant hypoosmolar plasma, (b) high urinary sodium excretion and high urinary osmolality, (c) normal renal, thyroid, and pituitary functions, (d) low urea and uric acid levels, (e) a lower anion gap, and (f) absence of diuretic intake [3].

As described before, the most common causes of SIADH were excluded by clinic, laboratory, and radiologic methods and were found as negative. 

In the differential diagnosis of the patient's febrile illness, we considered the epidemiologic context of infectious diseases. The most important infectious disease among them was Brucellosis in eastern part of Turkey where the disease is endemic [6].

Our patient also had clear evidence of brucellosis with nocturnal fever, arthralgia, relative leukopenia, elevated CRP levels, cholestatic hepatitis, splenomegaly, pericardial effusion, and finally seropositivity of Rose-Bengal test. 

Infectious disease-related SIADH is still poorly defined. Up to date, only one study of Brucellar SIADH has been published by Arabic authors [7].

Gastrointestinal symptoms are noted in 40% of patients with brucellosis. Occasionally, severe brucellar gastrointestinal localizations, such as brucellar hepatitis with cholestasis, may present with abdominal pain on the right upper quadrant as was seen in our case [8].

In a study from Eastern Turkey, elevation of the liver and cholestasis enzymes has been reported in cases of Brucellosis, and the deterioration of these tests was improved within several days by medical treatment [9].

Cardiac involvement of Brucellosis is very rare and mainly presents as endocarditis. Aortic and mitral valves are the most affected areas during Brucellosis. The most prominent characteristics of these patients are retrosternal pain, pericardial friction rub, and fever. Patients with pericarditis from brucellosis have a poorer prognosis than others [10, 11].

In a recent study from Turkey, incidence of pericarditis in patients with Brucellosis was reported as less than 1% [12]. In another retrospective study from Spain, Brucella pericarditis was reported in only 1.5% among 530 cases [13].

In the presented case, the patient had a mild retrosternal pain and had minimal electrocardiographic abnormality. Her echocardiographic examination showed minimal pericardial effusion without cardiac dysfunction. This cardiac complication which resolved by treatment, in connection with cholestasis and, SIADH is considered so rare in the English literature.

SIADH is becoming increasingly detected partly as a result of a higher awareness of its existence. Treatment must include correction of the underlying pathology. This case report indicates that Brucellosis may be associated with SIADH, cholestatic hepatitis, and pericardial effusion. Zoonotic causes of SIADH (zoonotic SIADH) should be considered in differential diagnosis of hyponatremia especially in cases of coexistent cholestasis in endemic areas.
